# Hybrid Silsesquioxane/Benzoate Cu_7_-Complexes: Synthesis, Unique Cage Structure, and Catalytic Activity

**DOI:** 10.3390/molecules27238505

**Published:** 2022-12-03

**Authors:** Alexey N. Bilyachenko, Victor N. Khrustalev, Evgenii I. Gutsul, Anna Y. Zueva, Alexander A. Korlyukov, Lidia S. Shul’pina, Nikolay S. Ikonnikov, Pavel V. Dorovatovskii, Dmitri Gelman, Elena S. Shubina, Georgiy B. Shul’pin

**Affiliations:** 1 A. N. Nesmeyanov Institute of Organoelement Compounds, Russian Academy of Sciences, Vavilov Str. 28, 119991 Moscow, Russia; 2 Peoples’ Friendship University of Russia (RUDN University), Miklukho-Maklay Str. 6, 117198 Moscow, Russia; 3 Zelinsky Institute of Organic Chemistry, Russian Academy of Sciences (RAS), Leninsky Prospect 47, 119991 Moscow, Russia; 4 Pirogov Russian National Research Medical University, Ostrovitianov Str. 1, 117997 Moscow, Russia; 5 National Research Center “Kurchatov Institute”, Akademika Kurchatova pl. 1, 123182 Moscow, Russia; 6 Institute of Chemistry, Edmond J. Safra Campus, The Hebrew University of Jerusalem, Jerusalem 91904, Israel; 7 Semenov Federal Research Center for Chemical Physics, Russian Academy of Sciences, Ulitsa Kosygina 4, 119991 Moscow, Russia; 8 Chair of Chemistry and Physics, Plekhanov Russian University of Economics, Stremyannyi Pereulok, Dom 36, 117997 Moscow, Russia

**Keywords:** metallasilsesquioxanes, cagelike compounds, benzoate ligands, oxidative catalysis, alkanes, alkyl hydroperoxide

## Abstract

A series of phenylsilsesquioxane-benzoate heptacopper complexes **1**–**3** were synthesized and characterized by X-ray crystallography. Two parallel routes of toluene spontaneous oxidation (into benzyl alcohol and benzoate) assisted the formation of the cagelike structure **1**. A unique multi-ligation of copper ions (from (*i*) silsesquioxane, (*ii*) benzoate, (*iii*) benzyl alcohol, (*iv*) pyridine, (*v*) dimethyl-formamide and (*vi*) water ligands) was found in **1**. Directed self-assembly using benzoic acid as a reactant afforded complexes **2**–**3** with the same main structural features as for **1**, namely heptanuclear core coordinated by (*i*) two distorted pentameric cyclic silsesquioxane and (*ii*) four benzoate ligands, but featuring other solvate surroundings. Complex **3** was evaluated as a catalyst for the oxidation of alkanes to alkyl hydroperoxides and alcohols to ketones with hydrogen peroxide and tert-butyl hydroperoxide, respectively, at 50 °C in acetonitrile. The maximum yield of cyclohexane oxidation products as high as 32% was attained. The oxidation reaction results in a mixture of cyclohexyl hydroperoxide, cyclohexanol, and cyclohexanone. Upon the addition of triphenylphosphine, the cyclohexyl hydroperoxide is completely converted to cyclohexanol. The specific regio- and chemoselectivity in the oxidation of *n*-heptane and methylcyclohexane, respectively, indicate the involvement of of hydroxyl radicals. Complex **3** exhibits a high activity in the oxidation of alcohols.

## 1. Introduction

Cagelike metallasilsesquioxanes (CLMSs) are an intensely studied class of metallacomplexes, providing a kaleidoscopic variety of molecular architectures [1,2,3,4,5,6,7,8,9,10,11] as well as demonstrating significant potential for various applications. The latter includes the design of molecular magnets (including single-molecule magnets and spin glasses) [12,13,14,15,16,17,18] and objects with photophysical properties (including the first example of a CLMS-based luminescent thermometer) [19,20,21,22]. For materials science, CLMSs were successfully applied for the production of functional coordination polymers [23,24,25,26], anode materials [27,28], biocompatible agents [29], and flame retardants [30]. Another important area of CLMSs investigations concerns their catalytic properties towards numerous important industrial processes [31,32,33,34,35,36]. Among recent results, we could mention CO_2_ cycloaddition [37], Chan–Evans–Lam coupling [38], biomass transformations [39], and oxidative amidation [40]. Considering the significant potential of copper-based CLMSs as catalysts [41,42,43], we pursue designing new types of these compounds. Numerous results pointed out the significant influence of systems used for synthesis/crystallization on the structural features of the CLMSs being formed. We could cite effects of “nuclearity shift”, e.g., Nb_2_ vs. Nb_4_ [44], Zn_7_ vs. Zn_8_ [45], Ni_5_ vs. Ni_6_ [46], Cu_4_Na_4_ vs. Cu_5_ [47]. Here a special versatility of copper-CLMSs should be emphasized as the most abundant representatives of this class of metallacomplexes. The kaleidoscopic variety of Cu-CLMSs included many attractive types of molecular geometry, e.g., Cubane [48], Cooling Tower [23], Fullerene-like [49], Tower of Pisa [50], Castel del Monte [51] and others [11]. Moreover, their nuclearity spanned from Cu_2_ [23,52] to Cu_24_ [49]. Our most recent results on the Cu-CLMS synthesis, structure determination, and catalytic evaluation are presented below.

## 2. Experimental

### 2.1. General Experimental Considerations

All reagents were purchased from the usual suppliers (Sigma, Fluka, St. Louis, MO, USA) and used without further purification. Elemental analyses were carried out with an XRF spectrometer VRA-30. IR spectra of the compounds (KBr pellets) were measured on a Shimadzu IR Prestige 21 FT-IR Spectrophotometer equipped with an MCT detector using a Miracle single reflection ATR unit by Pike. Signals observed (Figures S2–S4): 1600–1400 cm^−1^ (νC=C, νC=N), 1120 cm^−1^ (νPh–Si), 940–1100 cm^−1^ (νasSi–O, νasSi–O–Si), 900 cm^−1^ (νasSi–O in Si–O–M fragment), 720–680 cm^−1^ (σC–H of mono-substituted phenyl group). The rest of the signals are attributed to the presence of additional solvating ligands (pyridine/DMF in **1**, DMSO in **2**). UV-Vis spectra (Figures S5–S7, 10 mm optical path length) were recorded on a Cary 50 spectrophotometer. The d–d transitions are better resolved for **1** (Py solution) than for **2** and **3** (benzonitrile and acetone solutions, respectively) due to higher solubility in pyridine.

#### 2.1.1. Synthesis of **1**

1.00 g (5.05 mmol) of PhSi(OMe)_3_ and 0.28 g (7 mmol) of NaOH were heated at reflux in 35 mL of ethanol for 2 h. Then, 0.47 g (3.50 mmol) of CuCl_2_ was added, and the resulting mixture was stirred without heating for 24 h. Solution was separated from insoluble part by centrifugation and dried in vacuum. Resulting solid product was mixed with 45 mL of toluene and was heated at reflux for 28 h. Solution was left in contact with air for one week for crystallization, which was, however, unsuccessful. Then the solution was mixed with 15 mL of pyridine and dimethylformamide (1:1, *v*/*v*). Crystallization gave a crystalline material in 4–5 days, including single crystals that were used for X-ray diffraction analysis. The remaining part of the crystalline material was dried in vacuum to calculate yield.

Complex **1**. Anal. Calcd for (Ph_5_Si_5_O_10_)_2_Cu_7_(PhCOO)_4_: Cu, 19.33; Si, 12.20.

Found: Cu, 18.47; Si, 12.01. Yield: 0.11 g (10%).

#### 2.1.2. Syntheses of **2**–**3**

In a typical procedure, 1.00 g (5.05 mmol) of PhSi (OMe)_3_ and 0.28 g (7 mmol) of NaOH were heated at reflux in 30 mL of ethanol for 2 h. Then, 0.47 g (3.50 mmol) of CuCl_2_ was added, and the resulting mixture was kept at reflux for 12 h, cooled to room temperature, and mixed with 0.24 g (2 mmol) of benzoic acid. Solution was stirred without heating for 6 h, followed by centrifugation of precipitate. Filtrate of **2** was mixed with 15 mL of dimethylsulfoxide. Crystallization gave (in ~10 days for **2** and in 3–4 days for **3**) a crystalline material, including single crystals that were used for X-ray diffraction analysis. The remaining part of the crystalline material was dried in a vacuum to calculate yield.

Complex **2**. Anal. Calcd for (Ph_5_Si_5_O_10_)_2_Cu_7_(PhCOO)_4_: Cu, 19.33; Si, 12.20.

Found: Cu, 19.02; Si, 12.06. Yield: 0.41 g (36%).

Complex **3**. Anal. Calcd for (Ph_5_Si_5_O_10_)_2_Cu_7_(PhCOO)_4_: Cu, 19.33; Si, 12.20.

Found: Cu, 19.20; Si, 12.09. Yield: 0.62 g (54%).

### 2.2. X-ray Crystal Structure Determination

The single-crystal X-ray diffraction data for **1** were collected on a three-circle Bruker APEX-II CCD diffractometer (*T* = 100 K, *λ*(Mo*K*α)-radiation, graphite monochromator, *ω*- and *φ*-scanning mode). The data were indexed and integrated using the *SAINT* program [53] and then scaled and corrected for absorption using the *SADABS* program [54]. The single-crystal X-ray diffraction data for **2** were collected on a four-circle Rigaku Synergy S diffractometer equipped with a HyPix6000HE area-detector (*T* = 100 K, *λ*(Cu*K*α)-radiation, graphite monochromator, shutterless *ω*-scanning mode). The data were integrated and corrected for absorption by the *CrysAlisPro* program [55]. The single-crystal X-ray diffraction study of **3** was carried out on the ‘RSA’ beamline (*T* = 100 K, *λ* = 0.74500 Å) of the Kurchatov Synchrotron Radiation Source. In total, 720 frames were collected with an oscillation range of 1.0° in the *φ* scanning mode using two different orientations of the crystal. The semi-empirical correction for absorption was applied using the *Scala* program [56]. The data were indexed and integrated using the utility *iMOSFLM* from the CCP4 software suite [57,58]. For details, see Table S1.

The structures were solved by intrinsic phasing modification of direct methods [59] and refined by a full-matrix least-squares technique on *F*^2^ with anisotropic displacement parameters for all non-hydrogen atoms. One out of the three coordinated DMF ligands in **1** is disordered over two sites with the occupancies of 0.70:0.30. Two of the fourteen phenyl groups and all six coordinated ethanol ligands in **3** are disordered over two sites each with the occupancies of 0.60:0.40, 0.50:0.50, 0.60:0.40, 0.65:0.35, 0.50:0.50, 0.65:0.35, 0.55:0.45, and 0.65:0.35, respectively. The hydrogen atoms of the hydroxyl groups in **1**–**3**, as well as the coordinated water molecules in **1**, were objectively localized in the difference-Fourier maps and refined isotropically. The other hydrogen atoms were placed in calculated positions and refined within the riding model with fixed isotropic displacement parameters [*U*_iso_(H) = 1.5*U*_eq_(C) for the CH_3_ groups and 1.2*U*_eq_(C) for the other groups]. All calculations were carried out using the *SHELXTL* program [60,61].

Crystallographic data for **1**–**3** have been deposited with the Cambridge Crystallographic Data Center, CCDC 2216232-2216234, respectively. Copies of this information may be obtained free of charge from the Director, CCDC, 12 Union Road, Cambridge CB2 1EZ, UK (Fax: +44-1223-336033; e-mail: deposit@ccdc.cam.ac.uk or www.ccdc.cam.ac.uk).

## 3. Results and Discussion

### 3.1. Synthesis and Structure

**Scheme 1:**
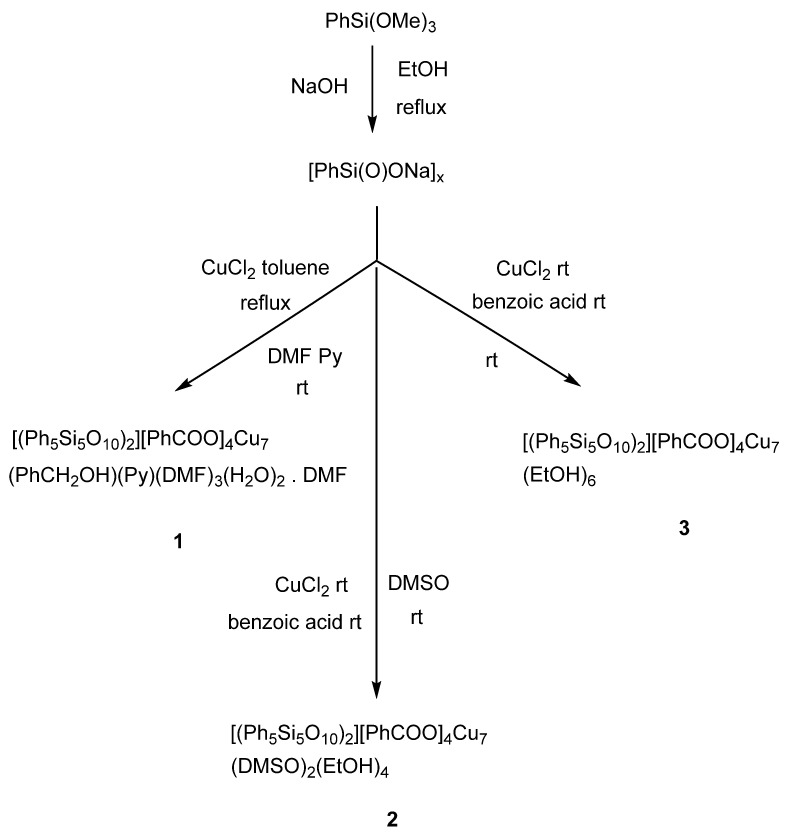
A general method for preparing copperphenylsilsesquioxane/benzoate complexes **1**–**3**.

**Figure 1:**
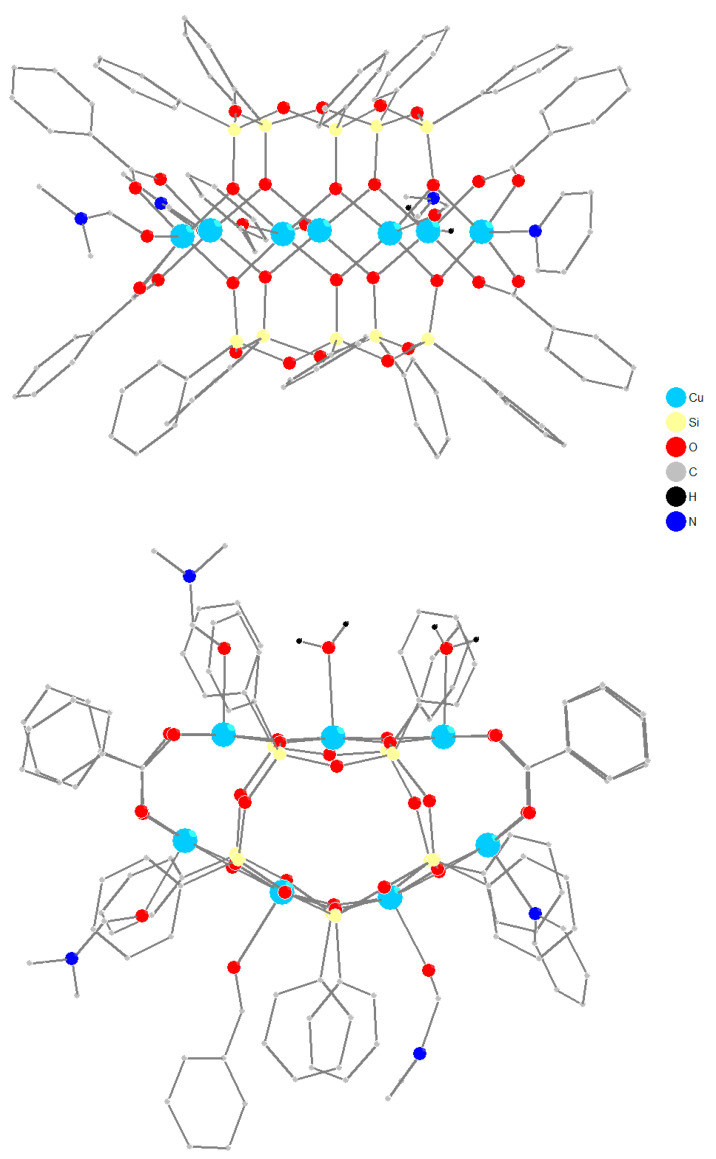
Side (**top**) and top (**bottom**) views of the molecular structure of **1**.

For the synthesis, a convenient scheme (Scheme 1) that included alkaline hydrolysis of RSi(OMe)_3_ [62,63] and subsequent reaction of the intermediate siloxanolate [PhSi(O)ONa]_x_ building blocks with copper(II) chloride in ethanol solution was applied. Toluene was chosen as an additional system component for the synthesis/crystallization. Noteworthy, despite several toluene-assisted syntheses of CLMSs, reported earlier [5,64,65], CLMS structures, including toluene as solvating ligand, remain quite rare [66,67,68]. Crystallization of potential CLMS single crystals in the native system failed; thus, an additional portion of solvents with a high coordination ability (pyridine and dimethylformamide) was used (Scheme 1). This method allowed us to successfully isolate (in a 10% yield) a product **1** of [(Ph_5_Si_5_O_10_)_2_][PhCOO]_4_Cu_7_(PhCH_2_OH)(Py)(DMF)_3_(H_2_O)_2_ · DMF composition (Figure 1).

**Figure 2:**
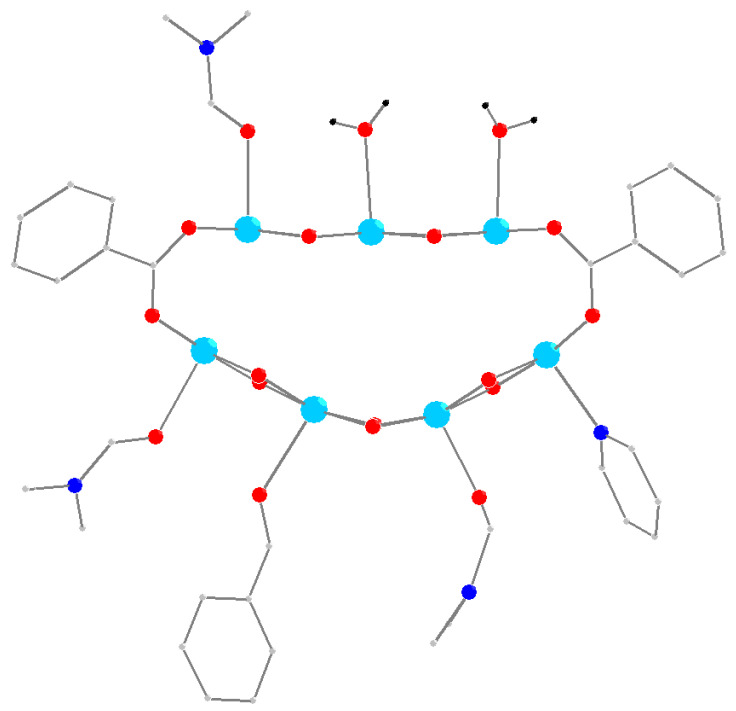

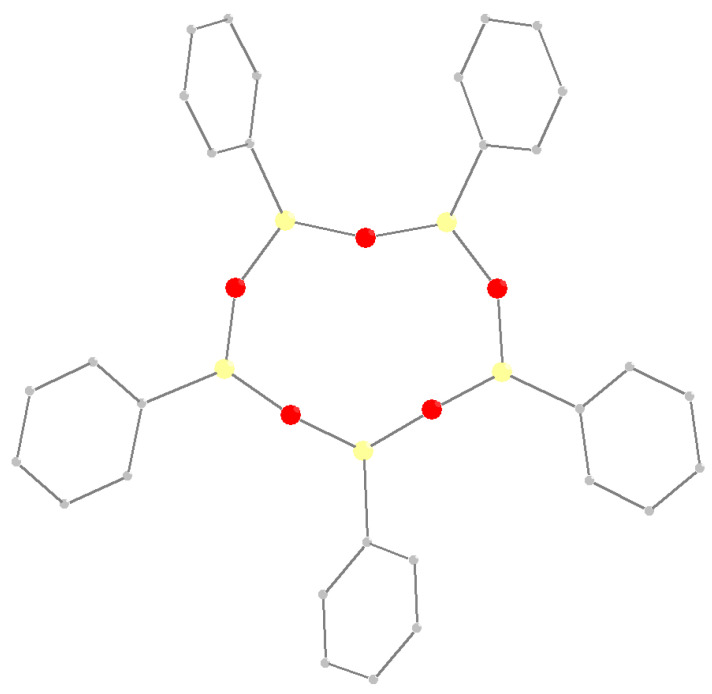
(**Top**) Representation of copper ions location and their multi-ligation style in **1**. (**Bottom**) Distorted pentameric siloxane ligand in **1**. The color code is the same as in Figure 1.

Complex **1** exhibits a set of unprecedented structural features. First of all, to the best of our knowledge, **1** is only the second observation of heptanuclear CLMS [69]. Second, the self-assembly of **1** is assisted by two distinct routes of spontaneous oxidation of toluene—(*i*) to benzyl alcohol and (*ii*) to benzoate. Both benzyl alcohol and benzoate favor the formation of **1** by playing the role of additional ligands. Several examples of spontaneous alcohol oxidations, giving unexpected carboxylic components of cage metallasilsesquioxanes, were reported for Cu-CLMSs [43,51,70]. To the best of our knowledge, the only example of methyl group hydroxylation-assisted CLMS self-assembly was reported by us for the oxidation of neocuproine into the corresponding diol [71]. Thus, the oxidation of toluene to benzyl alcohol is quite an unusual observation for CLMS chemistry. In sum, both toluene oxygenates (benzoates and benzyl alcohol) coordinate copper ions in **1**. To the best of our knowledge, such a combination of oxidized toluene derivatives has never been found for any metallacomplexes to date. More than that, six different ligand types (silsesquioxane, benzoate, benzyl alcohol, pyridine, dimethylformamide, and water) in **1** is a record high multiligation of metallacomplex ever observed. Such a diversity of ligands provoked two additional features of the cage structure of **1**. First of all, the location of copper ions is unusual for CLMSs. It represents (*i*) three copper ions in an almost linear (178.04(2)º) fragment and (*ii*) four copper ions in a circular arc fragment (Figure 2 top). Second, pentameric ‘heart-like’ (Ph_5_Si_5_O_10_) silsesquioxane ligands in **1** (Figure 2 bottom) are significantly distorted as compared to earlier reported circle-like geometry [16,18,46,72]. We suppose that an equilibrium mixture of various cyclic silanolate (x = 3, 4, 6, 8…) is initially generated in the hydrolysis of [PhSi(O)ONa]_x_ as intermediates, whereas the pentameric form of complex **1** is organized via their concerted coordination to copper centers.

**Figure 3:**
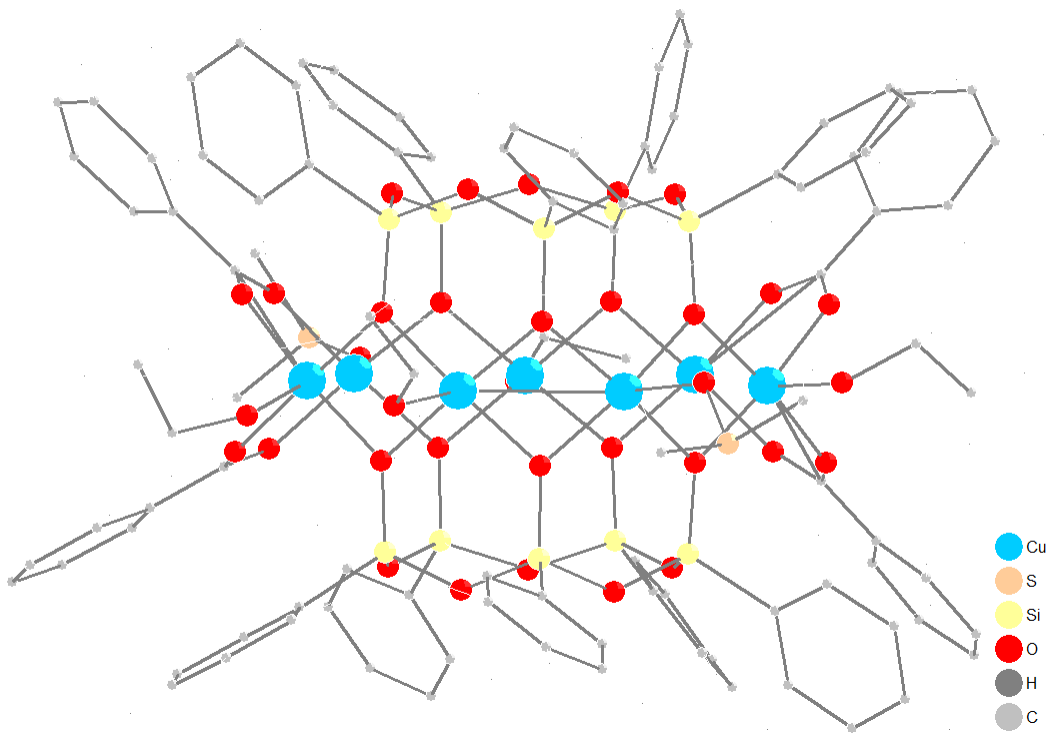
Side view of the molecular structure of **2**.

On the other hand, the multiplicity of the ligand set (as well as the necessity of preliminary toluene oxidations) prevents a high yield of complex **1**. To overcome this issue, we performed additional reaction runs with a precise stoichiometric ratio of key components according to the as-received composition of **1** (10Si:7Cu:4benzoates). Using benzoic acid as a reagent (Scheme 1), two analogs of complex **1** were successfully prepared. More precisely, complex **2** (Figure 3) of [(Ph_5_Si_5_O_10_)_2_][PhCOO]_4_Cu_7_(DMSO)_2_(EtOH)_4_ composition was isolated in a 36% yield from ethanol/dimethylsulfoxide solvate system. Furthermore, complex **3** (Figure S1) of [(Ph_5_Si_5_O_10_)_2_][PhCOO]_4_Cu_7_(EtOH)_6_ composition was obtained in a 54% yield from an ethanol-only solvate system.

Surprisingly, despite the different ligands coordinated to the copper atoms, compounds **2** and **3** are isostructural. A change in solvating surroundings in compounds **2**–**3** compared to complex **1** does not influence the general type of cage fragment based on the heptanuclear core bound by two distorted pentameric cyclic silsesquioxane and four benzoate ligands. Nevertheless, some structural deviations could be mentioned. First, due to steric reasons, the three linearly disposed copper atoms are capable of coordinating only two bulk ligands. Hence, in molecules **2** and **3**, these copper atoms coordinate the dimethylsulfoxide and ethanol and two ethanol solvent molecules, respectively. It is important to point out the fact that, both in **2** and **3**, the tetraccoordinated “naked” copper atom forms the intramolecular non-covalent Cu⋅⋅⋅H interaction with the hydrogen atom of the methyl group of the neighboring coordinated ethanol molecule at the distance of 2.987 and 3.044/3.090 Å, respectively. Second, the distance between opposite carboxylate carbon atoms in compound **1** is equal to 10.291(6) Å and 10.313(5) Å, in compound **2**—10.1291(14) Å and 10.3211(13) Å, in compound **3**—10.1292(19) Å and 10.3447(15) Å.

In the context of functional properties, a representative compound **3** (available in a higher yield) has been tested as a homogeneous catalyst toward the oxidative functionalization of hydrocarbons (cyclohexane, *n*-heptane, methylcyclohexane) and alcohols.

### 3.2. Complex ***3*** as a Catalyst in Oxidations with Peroxides

In recent years, the search for fundamentally new ways of involving hydrocarbons in chemical transformations has become a topic of great importance. There is growing interest in new catalytic systems capable of efficiently and selectively functionalizing alkanes under mild conditions. Certain metal complexes are known to catalyze reactions of hydrocarbons and alcohols occurring with the functionalization of C-H bonds [73,74,75,76,77,78,79]. Hydrogen peroxide and organic hydroperoxides are often used as oxidants in these processes. Copper complexes have systematically demonstrated high activity in these oxidation processes [80,81,82,83,84,85]. Recently, we also have found several copper complexes exhibited high efficiency in the oxidation of organic compounds with peroxides [86,87,88,89,90,91,92].

### 3.3. Oxidation of Alkanes

We discovered that complex **3** exhibits high catalytic activity in the oxidation of saturated hydrocarbons. The data obtained in the oxidation of cyclohexane with hydrogen peroxide for complex **3** showed that in the presence of HNO_3_, the reactions proceed much faster, which is consistent with our previous studies of oxidative processes using copper complexes as catalysts [68,69,70,86,88,89,90,91]. Nitric acid is a co-catalyst in these reactions. If the reaction is carried out in the absence of nitric acid, only the decomposition of hydrogen peroxide (catalase activity) is observed, and the yield of the reaction products was about 0.003 M (0.6%)

**Figure 4:**
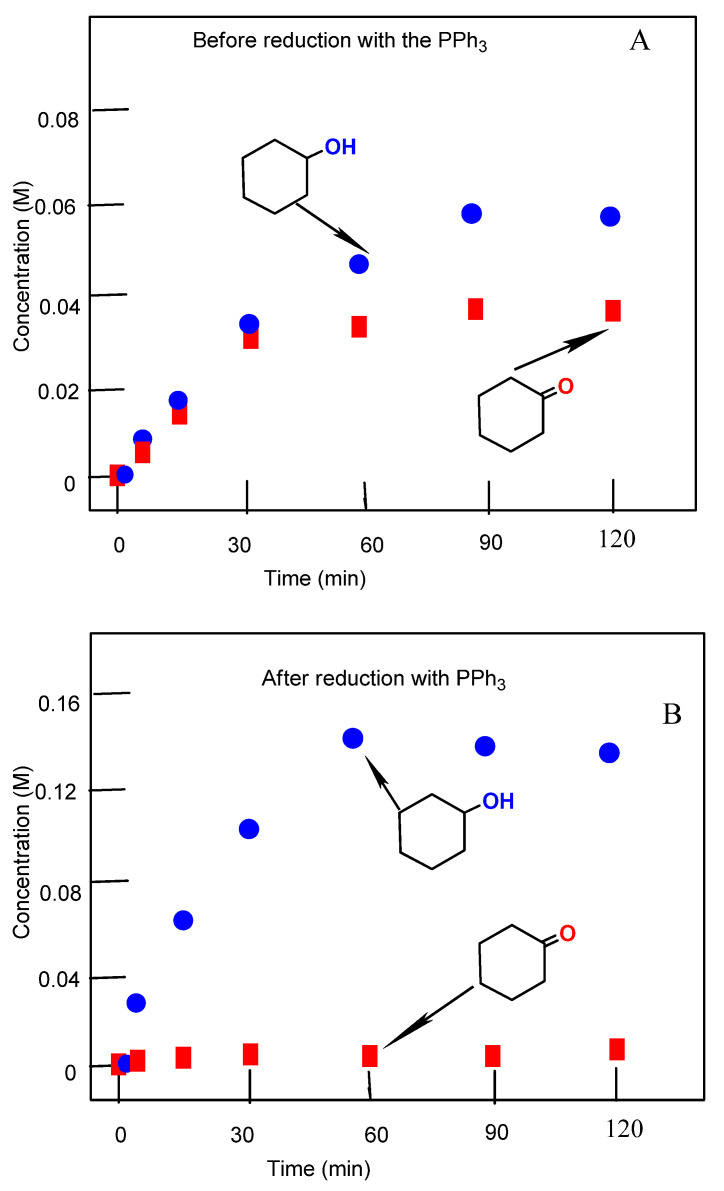
Accumulation of cyclohexanol and cyclohexanone in the oxidation of cyclohexane (0.46 M; 0.125 mL in 2.5 mL total volume) with hydrogen peroxide (2.0 M, 0.32 mL 50% aqueous in 2.5 mL total volume) catalyzed by compound **3** (5 × 10^−4^ M; 3 mg in 2.5 mL total volume) in the presence of HNO_3_ (0.05 M; 0.5 mL stock solution in 2.5 mL total volume) in MeCN at 50 °C. Concentrations of cyclohexanone and cyclohexanol were determined before the reduction of the solid PPh_3_ (**A**). The same reaction after reducing the aliquots with solid PPh_3_ (**B**).

Oxidation of cyclohexane with hydrogen peroxide according to gas chromatography results in the formation of a mixture of cyclohexanol and cyclohexanone. Accumulations of the products are presented in Figure 4A,B. The ratio of cyclohexanone to cyclohexanol for complex **3** is about 1 (0.017 M/0.016 M after 60 min.). Adding triphenylphosphine to the reaction mixture according to the method proposed previously by one of us [93,94] gives rise to a sharp increase in the concentration of cyclohexanol and a noticeable decrease in the amount of cyclohexanone, as shown in Figure 4B. (The ratio of cyclohexanone to cyclohexanol after adding triphenylphosphine is about 0.04). Since the main product of the oxidation reaction is alkyl hydroperoxide, this result can be explained by the fact that in the absence of triphenylphosphine, the alkyl hydroperoxide present in the reaction mixture decomposes to form ketone and alcohol in comparable amounts both in the reaction solution and in the GC injector. Triphenylphosphine reduces an alkyl hydroperoxide to corresponding alcohol; therefore, we see a sharp increase in the amount of alcohol and a decrease in the amount of ketone on the chromatogram. The total yield of alcohol and ketone in the oxidation of cyclohexane catalyzed by complex **3** after 2 h is 32%, and TON is 290.

**Figure 5:**
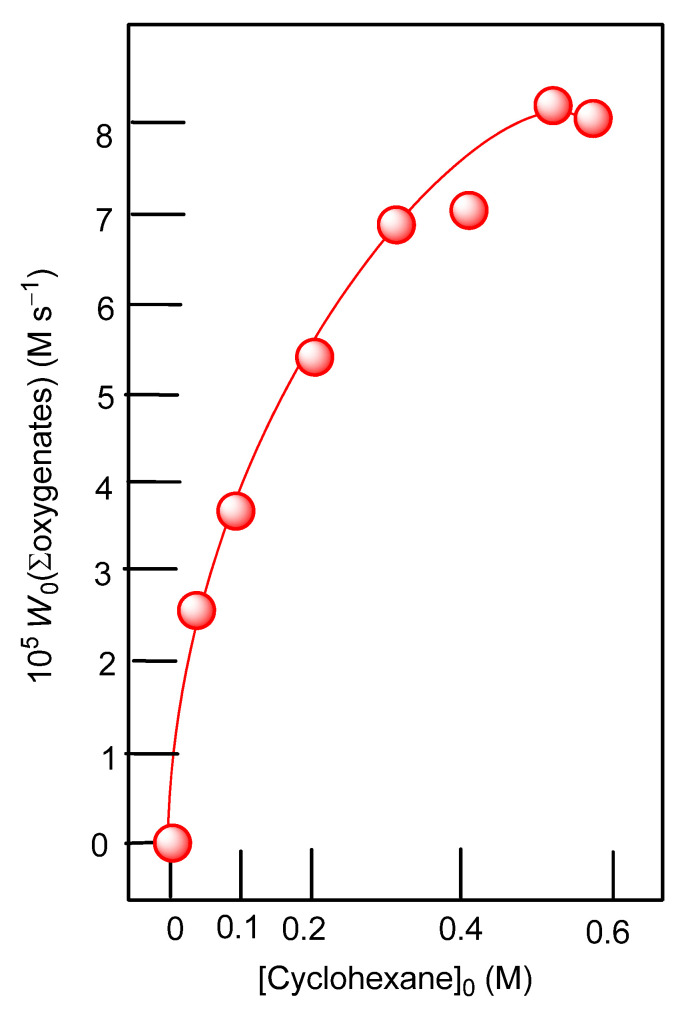
Dependence of the initial rate of oxygenate (sum cyclohexanol + cyclohexanone) formation *W*_0_ for complex **3** on the initial concentration of cyclohexane.

**Figure 6:**
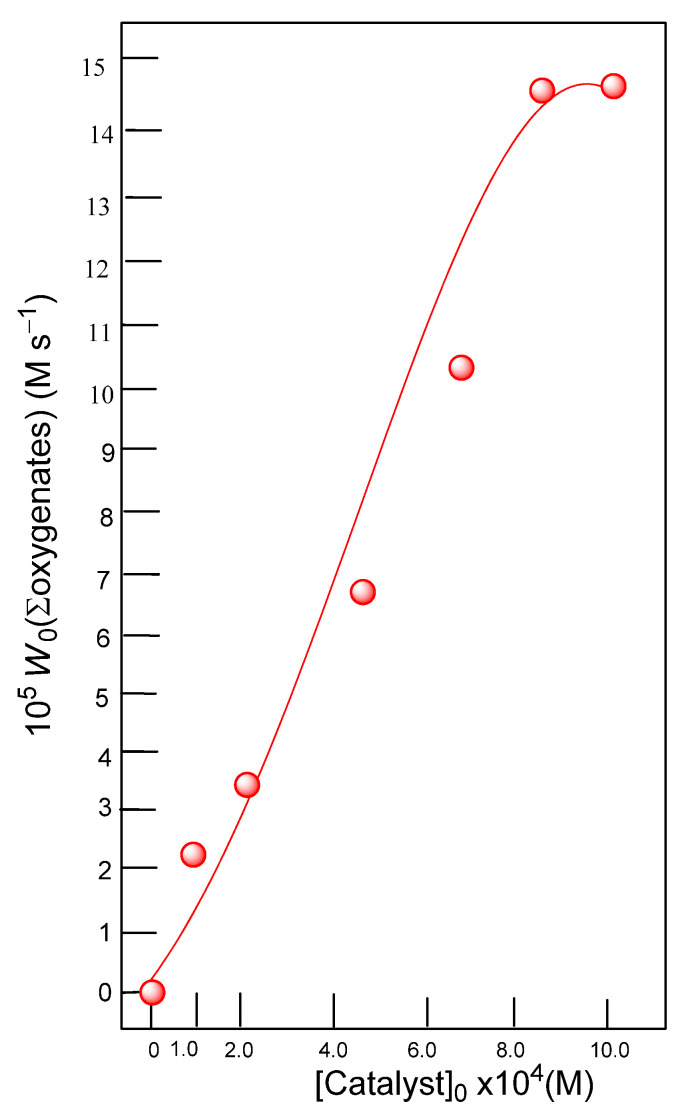
Dependence of the initial rate of oxygenate (sum cyclohexanol + cyclohexanone) formation *W*_0_ for complex **3** on the initial concentration of catalyst.

Dependences of initial rates of cyclohexane oxidation with hydrogen peroxide on initial concentrations of cyclohexane and catalyst are presented in Figure 5 and Figure 6, respectively. To assess the reactivity of a species arising from the decomposition of hydrogen peroxide under the action of complex **3** and inducing alkane oxidation, the influence of the alkane concentration on the initial rate of its oxidation was studied (see Figure 5). The form of the dependence is consistent with the assumption of the competitive interaction of the solvent acetonitrile and alkane (RH) with the intermediate species of an oxidizing nature. As the alkane concentration increases, the rate of its oxidation reaches its maximum limiting value.

The oxidation of *n*-heptane with hydrogen peroxide catalyzed by complex **3** was investigated. The results are given below (initial concentrations: *n*-heptane (0.5 M; 0.12 mL; H_2_O_2_ 1.5 M, 0.32 mL 50% aqueous; catalyst 5 × 10^−4^ M, 3 mg; HNO_3_ 0.05 M, 0.05 mL stock solution; CH_3_CN up to 2.5 mL). After two hours, the following concentrations of (M) alcohol isomers were obtained (after reduction with triphenylphosphine, the yield of oxidation products—17%; TON = 170):

C (1) 0.007M; C (2) 0.026M; C (3) 0.026M: C (4) 0.012M. 

These values give a series of selectivity: C (1):C (2):C (3):C (4) = 1.0:5.4:5.4:4.8.

The selectivity parameters for the oxidation of methylcyclohexane were also obtained: 1°:2°:3° = 1.0:6.4:18.6 (under the same conditions, the yield of oxidation products are 18%; TOH = 180 (mainly alcohols, ketones in trace amounts). The selectivity parameters measured in the oxidation methylcyclohexane are close to the parameters typical for the reactions of alkanes with hydroxyl radicals [95,96]. However, the selectivity parameters obtained for *n*-heptane somewhat exceed the typical selectivity parameters for the oxidation of *n*-heptane with the participation of hydroxyl radicals. This suggests that other oxidizing species also take part in the process. 

We have included data from other publications to show that our new Cu complex is a very efficient catalyst in the oxidation of alkanes and is on par with other copper-containing catalysts (Table 1). The difference between them is noticeable—the maximum yields of oxidation products are obtained in a shorter time since the reaction rate is higher, apparently due to the influence of ligands

### 3.4. Oxidation of Alcohols

**Figure 7:**
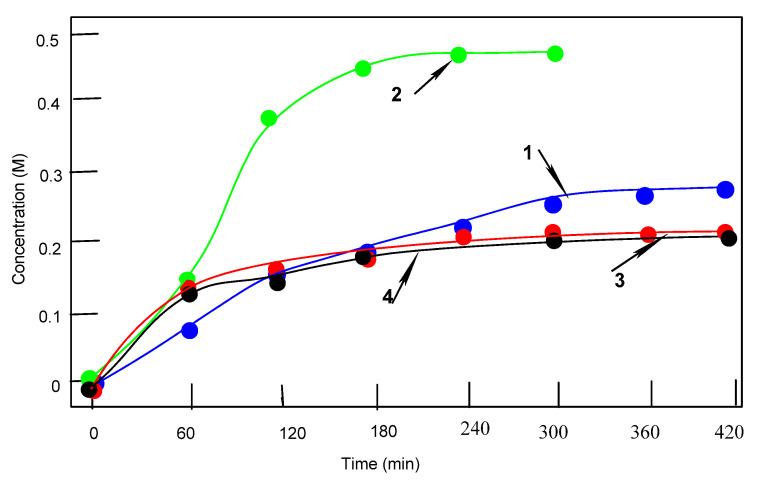
Accumulation of cyclohexanone (yield = 40%, TON = 400) in oxidation of cyclohexanol (0.5 M; 0.14 mL in 2.5 mL total volume; blue—curve 1), acetophenone (yield = 90%, TON = 900) in oxidation of phenylethanol (0.5 M; 0.16 mL in 2.5 mL total volume; green—curve 2), 2-heptanone (yield = 40%, TON = 400) in oxidation of 2-heptanol (0.5 M; 0.14 mL in 2.5 mL total volume red—curve 3) and 2-hexanone(yield = 40%, TON = 400) in oxidation of 2-hexanol (0.5 M; 0.16 mL in 2.5 mL total volume; black—curve 4) with *tert*-butyl hydroperoxide (1.5 M; 0.36 mL 70% aqueous in 2.5 mL total volume) catalyzed by compound **3** (5 × 10^−4^ M; 3 mg) in the absence of HNO_3_ in MeCN at 50 °C.

The accumulation of products in the reactions of aliphatic and aromatic alcohols with *tert*-butyl hydroperoxide is shown in Figure 7. They give ketones as oxidation products in high yields. It should be noted that these reactions do not require the addition of nitric acid. However, such reactions with hydrogen peroxide have very low efficiency. 

## 4. Conclusions

The synthesis of copperphenylsilsesquioxane mediated by an unusual spontaneous two-way oxidation of toluene led to an unprecedented cage structure **1,** including two specific ligands. These are derivatives of toluene oxidative transformation, i.e., mild one (benzylic alcohol) and harsh one (benzoate). Directed synthesis from ethanol/dimethylsulfoxide (complex **2**) or ethanol (complex **3**) media using benzoic acid as reactants afforded two complexes with cage structures analogous to compound **1**. Features of these cagelike compounds include unusual heptacopper nuclearity supported by the presence of two distorted (heart-like) pentameric silsesquioxane and four benzoate ligands. Such mixed ligand surrounding allowed us to observe two types of copper ions’ location: three ions as a linear fragment while the rest four in a circular arc unit. Compound **3** is a powerful catalyst for the efficient oxidation of alkanes with peroxides. Data on the selectivity of oxidation and the nature of the dependence of the initial rate of cyclohexane oxidation on the initial concentration of hydrocarbon indicate that the reaction proceeds with the participation of hydroxyl radicals and alkyl hydroperoxides are formed as the main primary products. Further investigations of the implementation of carboxylic reagents for the synthesis of metallasilsesquioxane cages in the design of catalytically active compounds are underway in our groups.

## Figures and Tables

**Table 1:** Comparison of catalytic efficiency of copper-based catalysts including complex **3**.

Catalytic System	Total Yield of Oxidation Products (%)	Time (Hours)	TON
Complex **3** (5 × 10^−4^ M) this work	32	1	290
Cu_2_Fe_5_-germsesquioxane [89]	28	1	260
Cu_9_Na_6_-silsesquioxane [26]	21	5.0	193
Cu(OAc)_2_ [97]	7.6	2.0	
Cu(NO_3_)_2_•3H_2_O + HNO_3_ (results of the authors)	9	2.0	80
Cu(NO_3_)_2_•3H_2_O (results of the authors)	2	24.0	

## Data Availability

Not available.

## Supplementary Materials

The following supporting information can be downloaded at: https://www.mdpi.com/article/10.3390/molecules27238505/s1. Details of syntheses, catalyzed reactions, and X-ray diffraction studies (CCDC 2216232-2216234). Figure S1: Side view of molecular structure **3**. Figures S2–S4: IR spectra of **1**–**3**. Figures S5–S7: UVvis spectra of **1**–**3**. Table S1: Crystal data and structure refinement for **1**–**3**
