# Supplementary material for: Hybrid Silsesquioxane/Benzoate Cu7-Complexes: Synthesis, Unique Cage Structure, and Catalytic Activity
Source: Molecules. 2022 Dec 3;27(23):8505. doi: 10.3390/molecules27238505 (PMC9739484; doi:10.3390/molecules27238505)
Supplement: Supplementary file 1 [file molecules-27-08505-s001.zip › molecules-2030676-supplementary.pdf]

Supplementary Materials

General experimental considerations

All reagents were purchased from the usual suppliers (Sigma, Fluka) and used without further purification. Elemental analyses were carried out with an XRF spectrometer VRA-30. IR spectra of the compounds (KBr pellets) were measured on a Shimadzu IR Prestige 21 FT-IR Spectrophotometer equipped with an MCT detector using a Miracle single reflection ATR unit by Pike. Set of signals: 1600-1400 cm−1 (νC=C, νC=N), 1120 cm−1 (νPh–Si), 940–1100 cm−1 (νasSi–O, νasSi–O–Si), 900 cm–1 (νasSi–O in Si–O–M fragment), 720–680 cm−1 (σC–H of mono-substituted phenyl group). Figure S2 is to exemplify a general view of spectra (see below). UV-Vis spectra (10 mm optical path length, acetone solution) were recorded on a Cary 50 spectrophotometer. Figure S3 is to exemplify a general view of spectra (see below).

Synthesis of 1

1.00 g (5.05 mmol) of $PhSi(OMe)_3$ and 0.28 g (7 mmol) of NaOH were heated at reflux in 35 ml of ethanol for 2 h. Then, 0.47 g (3.50 mmol) of $CuCl_2$ was added and the resulting mixture was stirred without heating for 24 h. Solution was separated from insoluble part by centrifugation and dried in vacuum. Resulted solid product was mixed with 45 ml of toluene and was heated at reflux for 28 h. Solution was left in the contact of air for one week for crystallization, which was found unsuccessful. Then solution was mixed with 15 ml of pyridine and dimethylformamide (1:1, v:v), crystallization gave in 4-5 days a crystalline material, including single crystals that were used for X-ray diffraction analysis. The remaining part of the crystalline material was dried in vacuum to calculate yield.

Complex **1**. Anal. Calcd for $(Ph_5Si_5O_{10})_2Cu_7(PhCOO)_4$: Cu, 19.33; Si, 12.20.

Found: Cu, 18.47; Si, 12.01. Yield: 0.11 g (10%).

Syntheses of 2-3

In a typical procedure, 1.00 g (5.05 mmol) of $PhSi(OMe)_3$ and 0.28 g (7 mmol) of NaOH were heated at reflux in 30 ml of ethanol for 2 h. Then, 0.47 g (3.50 mmol) of $CuCl_2$ was added and the resulting mixture was at reflux for 12 h, cooled to room temperature and mixed with 0.24 g (2 mmol) of benzoic acid. Solution was stirred without heating for 6 h followed by centrifugation of precipitate. Filtrate of **2** was mixed with 15 ml of dimethylsulfoxide. Crystallization gave (in ~10 days for **2**, 3-4 days for **3**) a crystalline material, including single crystals that were used for X-ray diffraction analysis. The remaining part of the crystalline material was dried in vacuum to calculate yield.

Complex **2**. Anal. Calcd for $(Ph_5Si_5O_{10})_2Cu_7(PhCOO)_4$: Cu, 19.33; Si, 12.20.

Found: Cu, 19.02; Si, 12.06. Yield: 0.41 g (36%).

Complex **3**. Anal. Calcd for $(Ph_5Si_5O_{10})_2Cu_7(PhCOO)_4$: Cu, 19.33; Si, 12.20.

Found: Cu, 19.20; Si, 12.09. Yield: 0.62 g (54%).

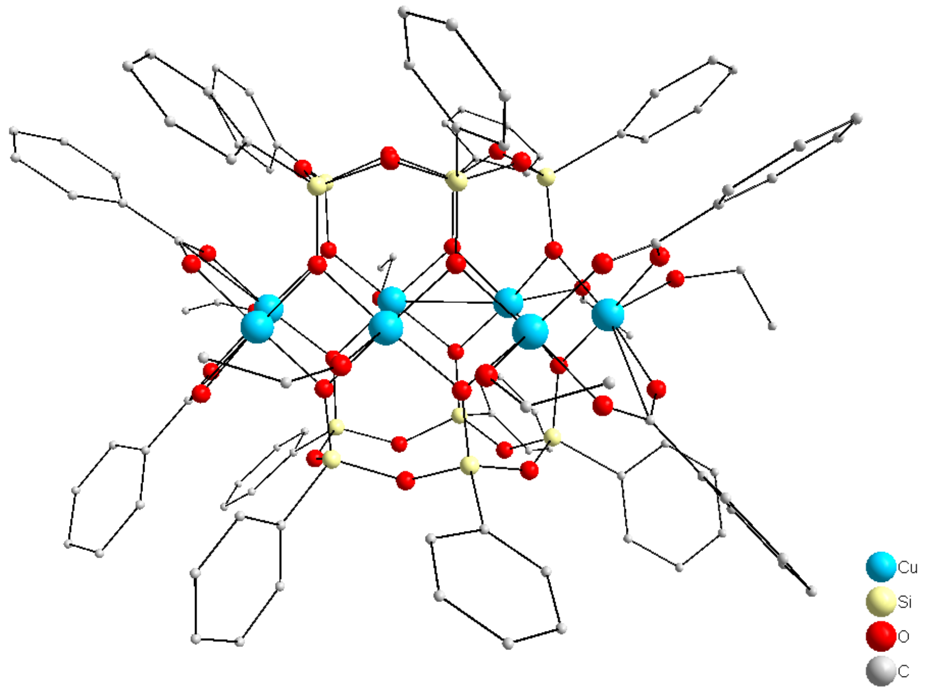

Figure S1. Side view of molecular structure **3**.

Table S1. Crystal data and structure refinement for **1-3**

Identification code	**1 • DMF**	**2**	**3**
Empirical formula	$C_{112}H_{115}Cu_7N_5O_{35}Si_{10}$	$C_{100}H_{106}Cu_7O_{34}S_2Si_{10}$	$C_{100}H_{106}Cu_7O_{34}Si_{10}$
Formula weight	2816.85	2641.72	2577.60
Crystal size, mm	0.20 × 0.25 × 0.30	0.06 × 0.09 × 0.15	0.05 × 0.11 × 0.16
Crystal system	Monoclinic	Monoclinic	Monoclinic
Space group	$P2_1/n$	*P*n	*P*n
a, Å	24.3418(17)	17.3652(2)	17.4559(17)
b, Å	16.7967(12)	18.8254(2)	18.5640(18)
c, Å	31.294(2)	18.5898(3)	18.5401(18)
α, deg.	90	90	90
β, deg.	94.713(2)	115.365(2)	115.027(14)
γ, deg.	90	90	90
V, Å^3	12751.7(16)	5491.28(15)	5443.8(11)
Z	4	2	2
Density (calc.), Mg/m^3	1.467	1.598	1.572
μ, mm^{-1}	1.316	3.545	1.739
F(000)	5780	2706	2642
Theta range, deg.	1.78 – 30.56	2.92 – 77.91	1.82 – 31.20
Index ranges	$-34 \le h \le 34$,<br>$-24 \le k \le 23$,<br>$-44 \le l \le 44$	$-21 \le h \le 21$,<br>$-19 \le k \le 23$,<br>$-23 \le l \le 22$	$-24 \le h \le 24$,<br>$-23 \le k \le 25$,<br>$-25 \le l \le 25$
Reflections collected	291773	85135	52702
Independent reflections, R_{int}	38925, 0.0877	21620, 0.0377	25285, 0.0342
Reflections observed	24114	19371	17507

R_1 / wR_2 ($I > 2\sigma(I)$)	0.0619 / 0.1469	0.0543 / 0.1401	0.0698 / 0.1659
R_1 / wR_2 (all data)	0.1145 / 0.1808	0.0620 / 0.1488	0.1021 / 0.1889
Goodness-of-fit on F^2	1.018	1.036	1.043
Extinction coefficient	–	–	0.0124(6)
T_{min} / T_{max}	0.667 / 0.746	0.606 / 0.800	0.765 / 0.888
$\Delta\rho_{max}$ / $\Delta\rho_{min}$, e·Å^{-3}	1.916 / -0.934	0.961 / -0.738	1.446 / -0.753

Oxidation of liquid alkanes:

Pyrex cylindrical vessels with vigorous stirring of the reaction mixute were used for the oxidation of alkanes with hydrogen peroxide were typically carried out in air in thermostated solution. Total volume of the reaction solution was 2.5 mL (**CAUTION**: the combination of air or molecular oxygen and H_2O_2 with organic compounds at elevated temperatures may be explosive!). Initially, a portion of 50% aqueous solution of hydrogen peroxide was added to the solution of the catalyst and substrate in acetonitrile. The aliquots of the reaction solution were analysed by GC (the instrument 3700, fused silica capillary column FFAP/OV-101 20/80 w/w, 30 m × 0.2 mm × 0.3 µm; helium as a carrier gas. Attribution of peaks was made by comparison with chromatograms of authentic samples.). Blank experiments with cyclohexane showed that, in the absence of catalyst **3**, no products were formed. Usually samples were analyzed twice, i.e. before and after the addition of the excess of solid PPh_3). This method was developed and used previously [S1-S2].

Alkyl hydroperoxides are transformed in the GC injector into a mixture of the corresponding ketone and alcohol. Due to this we quantitatively reduced the reaction samples with PPh_3 to obtain the corresponding alcohol. This method allows us to calculate the real concentrations not only of the hydroperoxide but of the alcohols and ketones present in the solution at a given moment.

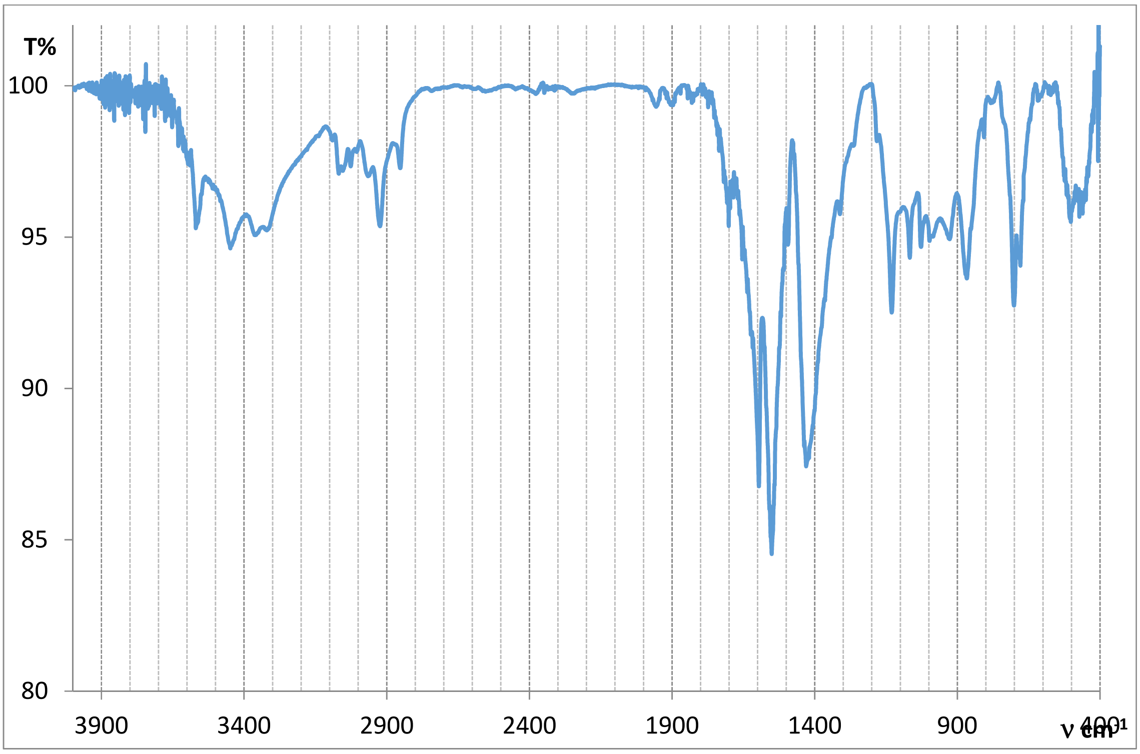

Figure S2. IR spectrum of **1**

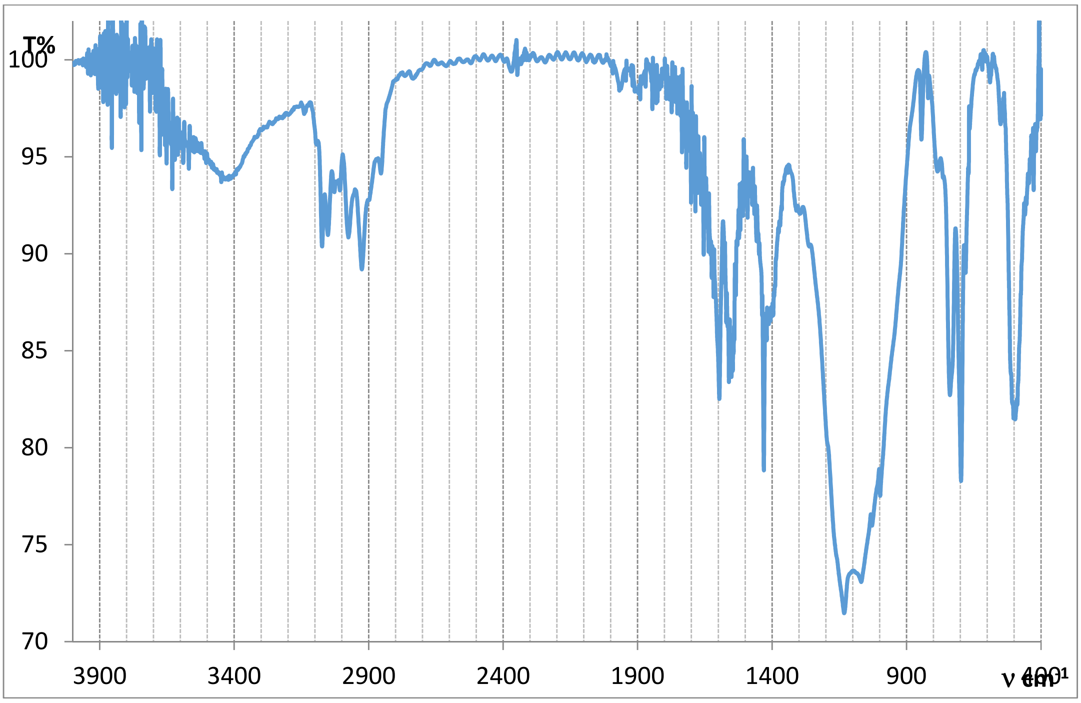

Figure S3. IR spectrum of **2**

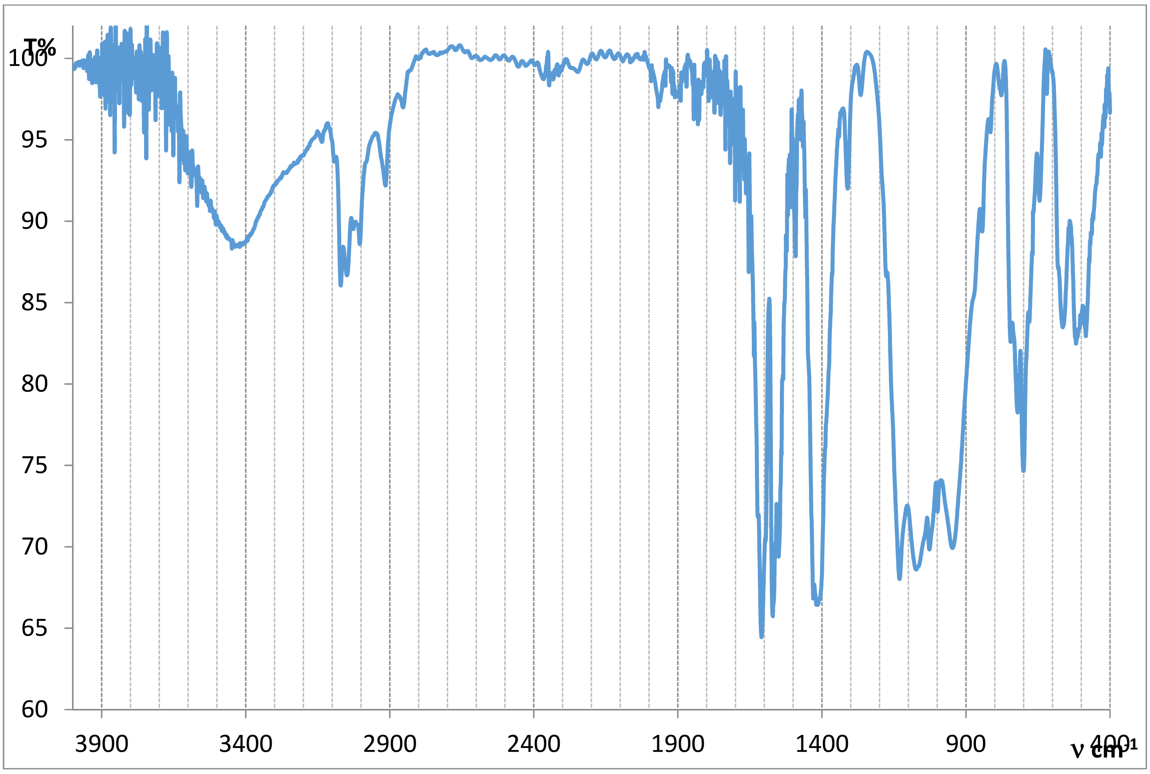

Figure S4. IR spectrum of **3**

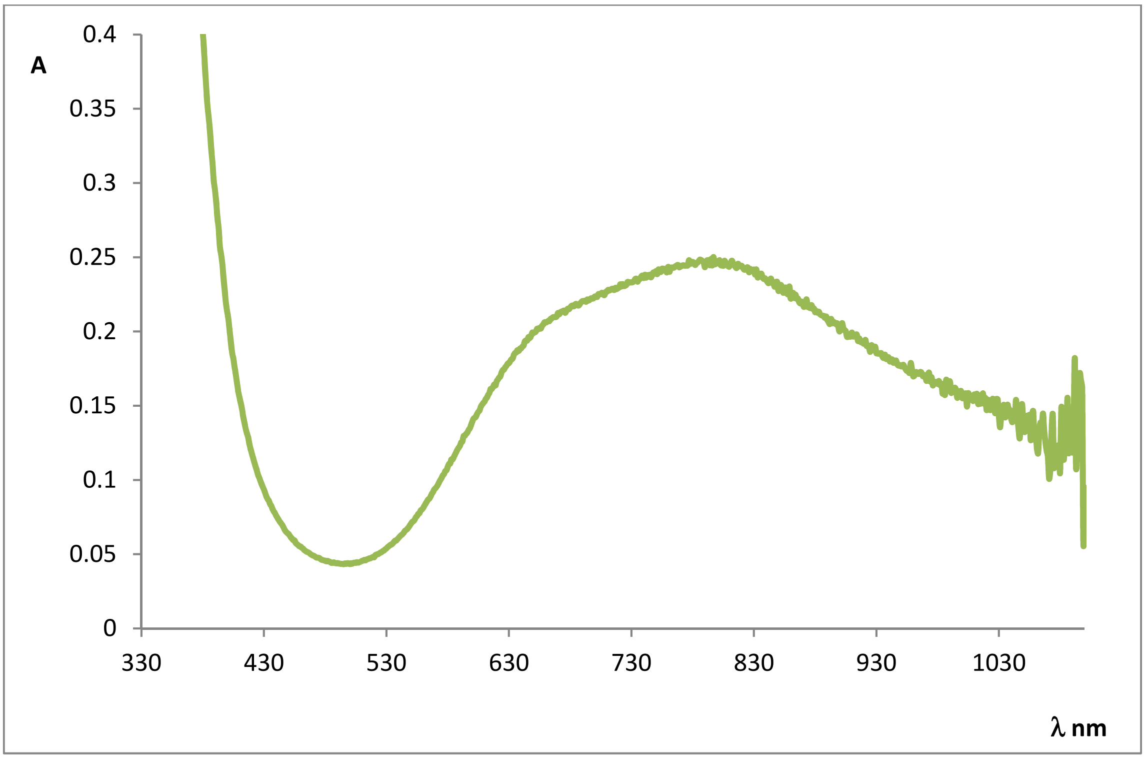

Figure S5. UV-vis spectrum of **1**

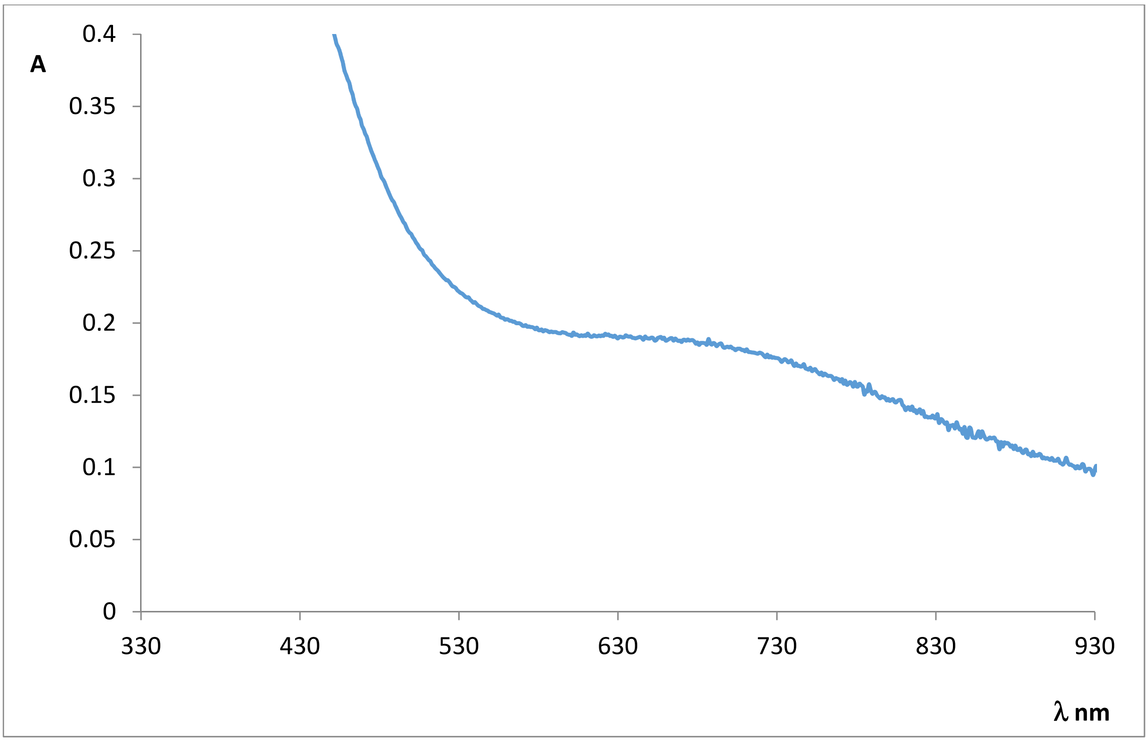

Figure S6. UV-vis spectrum of **2**

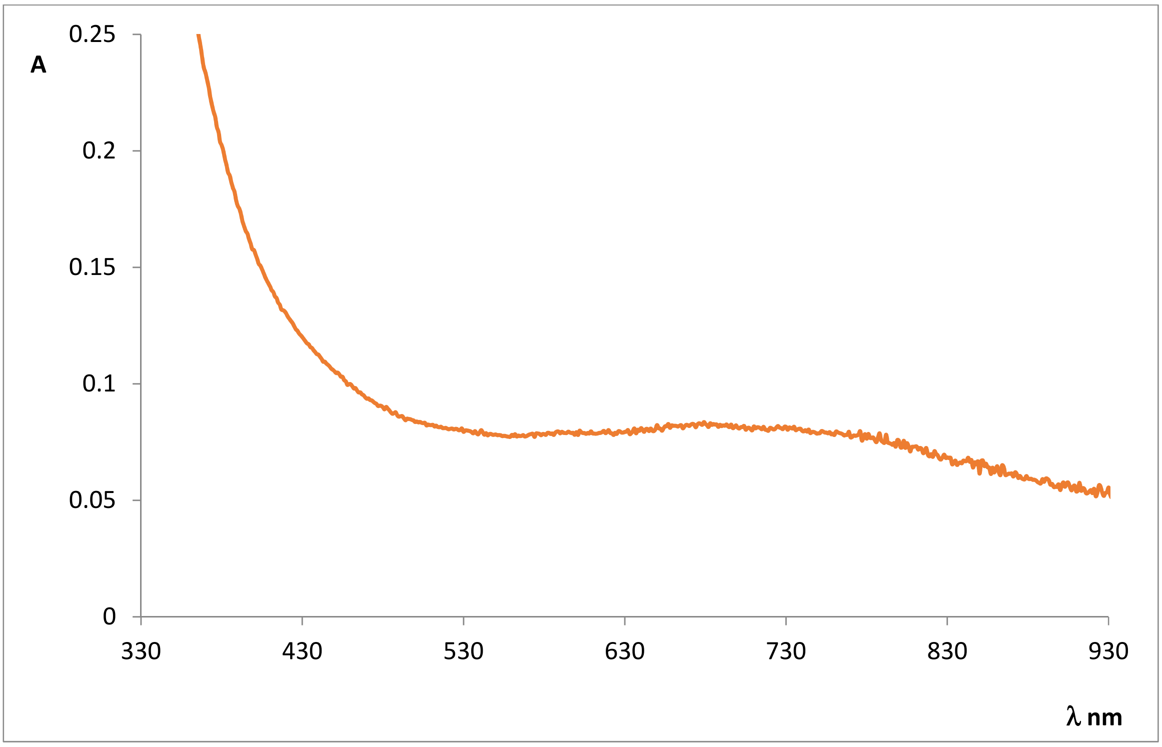

Figure S7. UV-vis spectrum of **3**

References

[1] Shul'pin, G.B. Metal-catalysed hydrocarbon oxygenations in solutions: the dramatic role of additives: a review. *J. Mol. Catal., A: Chem*. **2002**, *189*, 39–66

[2] Shul'pin, G.B.; Kozlov, Y.N.; Shul'pina, L.S.; Petrovskiy, P.V. Oxidation of alkanes and alcohols with hydrogen peroxide catalyzed by complex $Os_3(CO)_{10}(mu\text{-}H)_2$. *Appl. Organometal. Chem*. **2010**, *24*, 464–472
